# Changes in waist circumference and risk of all-cause and CVD mortality: results from the European Prospective Investigation into Cancer in Norfolk (EPIC-Norfolk) cohort study

**DOI:** 10.1186/s12872-019-1223-z

**Published:** 2019-10-28

**Authors:** Angela A. Mulligan, Marleen A. H. Lentjes, Robert N. Luben, Nicholas J. Wareham, Kay-Tee Khaw

**Affiliations:** 1 European Prospective Investigation into Cancer and Nutrition, Department of Public Health and Primary Care, Strangeways Research Laboratory, University of Cambridge, Worts Causeway, Cambridge, UK; 2 European Prospective Investigation into Cancer and Nutrition, MRC Epidemiology Unit, Cambridge Biomedical Campus, University of Cambridge, Cambridge, UK; 3 MRC Epidemiology Unit, Cambridge, Institute of Metabolic Science, Cambridge Biomedical Campus, University of Cambridge School of Clinical Medicine, Cambridge, UK; 4 EPIC, Department of Gerontology, Addenbrooke’s Hospital, School of Clinical Medicine, University of Cambridge, Cambridge, UK

**Keywords:** Waist circumference change, Weight change, All-cause mortality, CVD mortality, EPIC-Norfolk

## Abstract

**Background:**

Measures of abdominal adiposity are strongly associated with all-cause mortality and cardiovascular disease (CVD). However, data are limited and conflicting regarding the consequences of changes in body fat distribution. The main aims of this paper are to investigate the association between changes in waist circumference (WC) and all-cause and CVD mortality and to examine these changes in relation to concurrent changes in weight.

**Methods:**

The European Prospective Investigation into Cancer and Nutrition (EPIC-Norfolk) study recruited 25,639 participants between 1993 and 1997, aged 39–79, a number of whom also attended a second examination (1998–2000), and were followed up to 2016 for mortality. Participants were eligible for inclusion if they had WC, weight and height measurements at both time-points; those with a self-reported history of CVD or cancer, body mass index < 18.5 kg/m2 or missing data on covariates were excluded, leaving 12,337 participants for analyses. The median (IQR) follow-up time was 16.4 (15.7, 17.2) years. Hazard Ratios (HRs) for all-cause (2866 deaths) and CVD mortality (822 deaths), by categories of WC change, were determined using Cox proportional hazards analyses.

**Results:**

After multivariable adjustment, the HRs (95% CIs) for all-cause mortality for men and women with a WC gain (WCG) >  5 cm were 1.51 (1.29–1.75) and 1.25 (1.06–1.46) respectively. For CVD mortality in men and women with a WCG >  5 cm, the HRs were 1.84 (1.39–2.43) and 1.15 (0.85–1.55) respectively. In analyses of concurrent changes in WC and weight, the greatest risk (HRs) (95% CIs) in men occurred with weight loss and WCG: 1.80 (1.13–2.86) for all-cause and 2.22 (1.03–4.82) for CVD mortality. In women, the greatest risk for both all-cause (HR 1.50 (1.16–1.95)) and CVD mortality (HR 1.81 (1.15–2.85)) was observed in those with weight loss and maintenance of WC (WCM).

**Conclusions:**

Objectively measured WCG > 5 cm, was associated with subsequent higher total mortality risk and higher CVD mortality risk in men. Interventions focusing on preventing increase in central adiposity rather than lowering weight per se in later life may potentially have greater health benefits.

## Background

Obesity is a major risk factor for many chronic diseases, such as diabetes, cardiovascular disease (CVD) and certain cancer types, as well as mortality [1–3], and is commonly defined using body mass index (BMI) [4]. However, BMI does not take into account the distribution of the fat mass, which is of particular importance in older individuals, as the distribution of body fat changes with age [5]. Waist circumference (WC) strongly correlates with abdominal obesity and is a commonly used clinical measure of body fat distribution [6, 7]. Studies have shown that WC is associated with CVD risk [8–10] and CVD mortality [11]. WC has also been positively associated with all-cause mortality in a number of studies [12–18] with a few exceptions [19, 20]. The presence of central or abdominal obesity, as defined by a high WC, is one of five components used in the clinical diagnosis of the metabolic syndrome [21], which is associated with an increased risk of developing type 2 diabetes, CVD and subsequent mortality [22].

However, there are limited and conflicting data available regarding the consequences of changes in body fat distribution. A number of studies have found a gain in WC (WCG) to be predictive of subsequent all-cause and CVD mortality [23, 24] but a prospective cohort study found that WC loss (WCL) was associated with an increased risk of all-cause mortality, particularly for older adults [25]. However, no association was found between changes in WC and mortality in Swedish women [26], elderly Brazilians [27] or Iranian men [28].

Data pooled from 15 prospective studies that included 258,114 individuals, has found that a 1 cm increase in WC is associated with a 2% increased risk of future CVD [29]. In England, among women, the mean WC increased from 81.7 cm in 1993 to 88.1 cm in 2016, and the percentage with a very high WC (> 88 cm) rose from 26 to 46%; among men, the mean WC increased from 93.2 cm in 1993 to 97.0 cm in 2016, and the percentage with a very high WC (> 102 cm) increased from 20 to 34% [30].

We have recently reported that objectively measured weight loss (WTL), but not weight gain (WTG), was associated with subsequent higher mortality in healthy, middle-aged and elderly community-dwelling men and women, compared to those who maintained their weight (WTM) [31]. The main aim of this paper is therefore to investigate the association between changes in WC and all-cause and CVD mortality. A secondary aim is to examine these changes in relation to concurrent changes in weight as previous studies have tended to focus on either changes in WC or weight but not changes in both concurrently.

## Methods

### Study design

The European Prospective Investigation Into Cancer and Nutrition (EPIC) is large diet and cancer cohort [32]. The EPIC-Norfolk study participants, aged between 39 and 79 years, were recruited from 35 General Practitioners’ surgeries in the Norfolk area of East Anglia, from 1993 to 1997 [33]. General practice age sex registers act as a population sampling frame as practically all of the UK the population are registered with a general practice through the National Health Service. Many of the characteristics of this cohort at baseline were comparable to the UK national population, including age, sex and anthropometry measurements but this cohort had a lower proportion of current smokers [34].

Ethical approval for the study was given by the Norwich District Health Authority Ethics Committee and all participants gave written, informed consent.

### Main exposure: change in waist circumference

Of those who consented to take part in the study (*N* = 30,445), 25,639 attended a baseline health examination (1HE) (1993–1997) and 15,786 attended a second health examination (2HE) (1998–2000).

Trained nurses measured participants’ weight and height, with participants wearing light clothing and without shoes. Weight was measured to the nearest 100 g using digital scales (Salter, UK). Height was measured to the nearest 0.1 cm using a free-standing stadiometer. A D-loop non-stretch fibreglass tape was used to measure WC, which was measured at the smallest circumference between the ribs and iliac crest to the nearest 0.1 cm while the participant was standing erect, with the abdomen relaxed, the arms at the side, the feet together and at the end of a normal expiration, without the tape compressing the skin. The measurement was taken at the level of the umbilicus where there was no natural waistline. Body mass index (BMI) is defined as the body mass (weight) divided by the square of the height, and is expressed in kg/m2, and is commonly used to categorise individuals as underweight (< 18.5 kg/m2), normal weight (≥18.5 to < 25 kg/m2), overweight (≥25 to 30 kg/m2), or obese (≥ 30 kg/m2) [4].

Absolute change in WC (cm) was calculated as WC measured at 2HE minus WC at 1HE. Absolute weight change (kg) was calculated as weight measured at 2HE minus weight measured at 1HE. Participants were assigned to one of five WC change categories: > 5 cm loss, > 2.5 to ≤5 cm loss, ≤ 2.5 cm loss or gain (‘maintenance’, including zero change; considered the reference category), > 2.5 to ≤5 cm gain, > 5 cm gain. Annual WC change was calculated from the absolute difference in WC, divided by the participants’ time lapse between the health examinations (cm/y).

In order to study the possible association between changes in WC and changes in weight, participants were assigned to one of nine categories, based on a loss of > 2.5 cm or 2.5 kg, within 2.5 cm or 2.5 kg loss or gain (maintenance), or a gain of > 2.5 cm or 2.5 kg respectively. The nine categories were as follows: WTM (weight maintenance) and WCM (WC maintenance) (reference category); WTL (weight loss) and WCL (WC loss); WTL and WCM; WTL and WCG (WC gain); WTM and WCL; WTM and WCG; WTG (weight gain) and WCL; WTG and WCM; and WTG and WCG.

### Participant selection

**Fig. 1:**
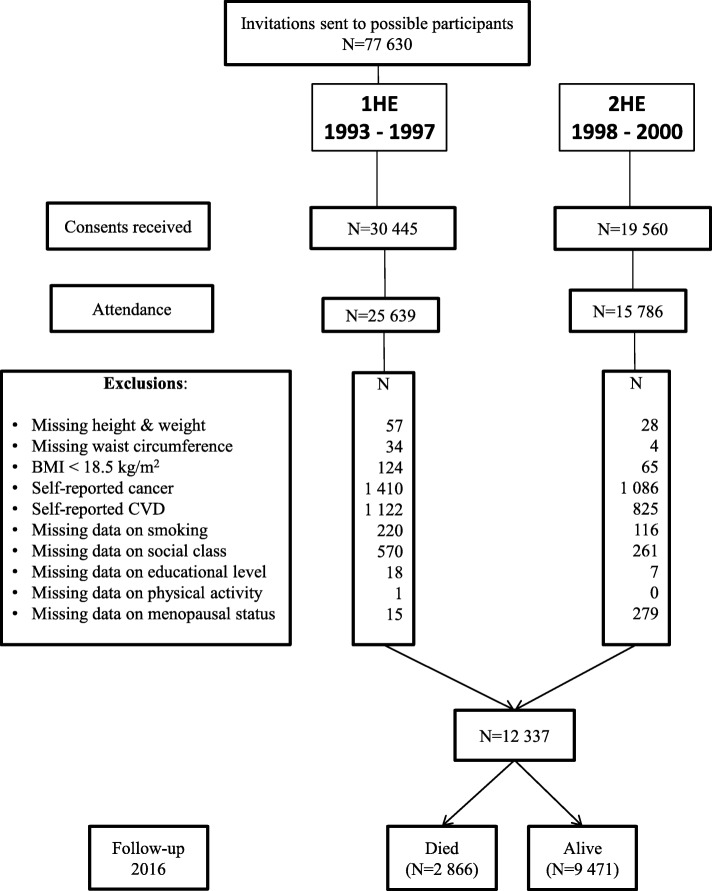
Study population and sample size eligible for mortality analyses

Measurements of WC, weight and height measurements at both time-points were essential for participant inclusion (*N* = 15,010). Participants were excluded from analyses if they were categorised as underweight (BMI < 18.5 kg/m^2^) or who self-reported CVD or cancer at either examination (*N* = 2095); those with missing data on adjustment variables (smoking, social class, educational level, physical activity, and menopausal status in women (*N* = 529) were also excluded. This left 12,337 participants for analyses (Fig. 1).

### Adjustment variables

Data from two self-administered Health and Lifestyle Questionnaires (HLQ1 and HLQ2), at 1HE and 2HE respectively, were used to create categories of a number of variables. Current smokers were defined as those currently smoking cigarettes, former smoker as being a smoker previously and non-smokers were those who never smoked (derived from HLQ1 and HLQ2). Self-reported physical activity (derived from HLQ1) was classified into four levels, using both occupational and leisure activity: inactive, moderately inactive, moderately active and active [35, 36]. Social class at 1HE was defined according to the Registrar General’s occupation-based classification scheme and categorised into the following six groups: professional, managerial and technical, non-manual skilled, manual skilled, partly skilled and unskilled [37]; for our analyses, these categories were combined to create 2 categories, non-manual (professional, managerial and technical, non-manual skilled) and manual (manual skilled, partly skilled and unskilled). Educational status at 1HE was categorised into four groups: degree or equivalent, A level or equivalent, O level or equivalent and less than O level or no qualification, corresponding to levels within the International Standard Classification of Education 1997 [38]. In our analyses, those with an educational level of O level and above were combined into one category. Menopausal status was categorised as premenopausal, early perimenopausal (< 1 year), late perimenopausal (1–5 years) or postmenopausal (> 5 years). Included adjustment variables were measured with HLQ1, with the exceptions of smoking status and menopausal status, which were measured with HLQ2. Participants were asked about their medical histories at both time-points. The diagnosis of chronic diseases such as heart attack, stroke and cancer were recorded as present when participants answered “yes” to question “Has a doctor ever told you that you have any of the following conditions?”

### Endpoints

All fatal events occurring between 1998 and 31st March 2016 were captured by the Office of National Statistics, United Kingdom. Death certificates were coded by nosologists according to the International Classification of Diseases (ICD). An underlying cause of death from cancer or CVD was defined by using the following ICD codes: cancer death (ICD9, 140–208 or ICD10 C00-C97), or CVD death (ICD9 400–438 or ICD10 I10-I79).

### Statistical analyses

Characteristics of the study population were summarised by WC change category, for continuous variables (mean and SD) and categorical variables (frequency and percentage). The follow-up time began at the 2HE and formed the underlying time variable; median (IQR) follow-up time was 16.4 (15.7, 17.2) years. Participants who died were censored at their date of death and those who did not die were censored at the end of follow-up (31st March 2016). Three Cox proportional hazards models were used to determine Hazard Ratios (HRs) for all-cause and CVD mortality by WC change category for men and women separately: age (continuous variable), (model 1); including BMI (continuous variable), WC (continuous variable), physical activity, social class, educational level, smoking (2HE) and menopausal status (2HE) in women (model 2); and including difference in weight (continuous variable) (model 3). The proportional hazards’ assumption was tested by including time interaction variables in the Cox regression models and we found that age violated our test (*P* < 0.0001). However, when the time interaction for age was included, only minimal changes to the HRs of WC change were observed, so results are shown with age adjustment alone. We also examined HRs by WC change category, separately in men and women, in subgroups, stratified by age, WC, smoking status, BMI, physical activity, educational level, social class, menopausal status in women, and after the exclusion of individuals who died within 3 or 5 years of the 2HE. The data were analysed using Stata 14 (STATA Corp., Texas, USA).

## Results

### Description of the cohort

Over a 3.5 year period, men had a mean WC and weight increase of 0.83 cm (SD 5.19) and 1.29 kg (SD 3.61) respectively; similar mean increases of 0.81 cm (SD 5.60) in WC and 1.38 kg (SD 4.14) in weight were found in women. The baseline characteristics of those who attended both 1HE and 2HE, before and after exclusions were applied were similar (Additional file 1: Table S1). However, the prevalence of self-reported CVD and the percentages of current smokers and physically inactive participants were higher in those who attended the 1HE only, compared to those who also attended the 2HE. Also, the percentage of deaths that occurred was lower in both men and women, after exclusion criteria were applied, with the highest percentage found in those who only attended the 1HE.

Table 1 describes the characteristics of men and women, by WC change category. WCM was observed in 40% of men and 38% of women. Participants with the greatest WCL were, on average, older and had the largest WC, weight and BMI at 1HE. Men with the greatest WCG were more likely to have a smaller WC at 1HE, whereas women with the greatest WCG had a similar WC at 1HE, compared to those in the WCM category. Current smokers at 2HE were more likely to have a WCL whereas former smokers, a WCG. Male non-manual workers were more likely to have a WCL > 5 cm whereas male manual workers were more likely to have a WCG > 5 cm. Participants with no qualifications were more likely to have a WCG > 5 cm. A greater percentage of deaths occurred in those with a WCL > 5 cm.

**Table 1:** Characteristics at 1st and 2nd health examinations of 5469 men and 6868 women, stratified by categories of change in waist circumference

	Categories of change in waist circumference (WC) (cm)											
	Loss > 5 cm		Loss > 2.5 and ≤ 5 cm		Loss or gain ≤2.5 cm		Gain > 2.5 and ≤ 5 cm		Gain > 5 cm		All	
*MEN, N (row %)*	641 (11.7)		656 (12.0)		2211 (40.4)		926 (16.9)		1035 (18.9)		5469	100.0%
WC at 1HE (cm)	99.0	9.2	96.8	9.1	95.3	8.9	93.8	8.6	92.4	9.7	95.1	9.3
WC at 2HE (cm)	91.0	9.2	93.2	9.1	95.4	8.9	97.6	8.6	100.5	10.0	96.0	9.6
WC change (cm)	−8.0	3.1	−3.7	0.7	0.1	1.4	3.8	0.7	8.1	2.9	0.8	5.2
Annual WC change (cm)	−2.3	1.0	−1.1	0.3	0.0	0.4	1.1	0.3	2.2	0.9	0.2	1.5
Age at 1HE (years)	59.6	9.3	59.7	9.0	58.8	8.9	58.7	8.9	58.6	8.7	58.9	8.9
Age at 2HE (years)	62.8	9.4	62.8	9.1	61.9	9.0	61.9	9.0	61.8	8.7	62.1	9.0
Age change (years)	3.2	0.8	3.1	0.8	3.1	0.8	3.2	0.8	3.3	0.8	3.2	0.8
Weight at 1HE (kg)	81.7	10.7	80.4	10.9	80.0	10.8	79.8	10.2	80.2	11.7	80.2	10.9
Weight at 2HE (kg)	79.0	10.5	79.9	11.0	81.0	11.2	82.4	10.7	84.4	12.8	81.5	11.5
Weight change (kg)	−2.7	3.6	−0.5	2.9	1.1	2.6	2.6	2.7	4.2	3.5	1.3	3.6
BMI at 1HE (kg/m^2^)	26.8	3.2	26.5	3.1	26.3	3.1	26.2	3.0	26.3	3.3	26.3	3.1
BMI at 2HE (kg/m^2^)	26.0	3.1	26.4	3.2	26.7	3.2	27.1	3.1	27.8	3.6	26.9	3.3
BMI change (kg/m^2^)	−0.8	1.2	−0.1	0.9	0.4	0.9	0.9	0.9	1.5	1.2	0.5	1.2
Education - 1HE												
None	172	26.8	164	25.0	573	25.9	248	26.8	287	27.7	1444	26.4%
O level and above	469	73.2	492	75.0	1638	74.1	678	73.2	748	72.3	4025	73.6%
Social class - 1HE												
Non-manual	412	64.3	414	63.1	1352	61.2	585	63.2	632	61.1	3395	62.1%
Manual	229	35.7	242	36.9	859	38.8	341	36.8	403	38.9	2074	37.9%
Smoking status - 1HE												
Current	66	10.3	60	9.2	185	8.4	86	9.3	110	10.6	507	9.3%
Former	321	50.1	341	52.0	1200	54.3	477	51.5	559	54.0	2898	53.0%
Never	254	39.6	255	38.9	826	37.4	363	39.2	366	35.4	2064	37.7%
Smoking status - 2HE												
Current	67	10.5	59	9.0	166	7.5	65	7.0	81	7.8	438	8.0%
Former	320	49.9	342	52.1	1221	55.2	498	53.8	589	56.9	2970	54.3%
Never	254	39.6	255	38.9	824	37.3	363	39.2	365	35.3	2061	37.7%
Physical activity - 1HE												
Inactive	193	30.1	160	24.4	601	27.2	233	25.2	257	24.8	1444	26.4%
Moderately inactive	163	25.4	196	29.9	529	23.9	216	23.3	249	24.1	1353	24.7%
Moderately active	156	24.3	159	24.2	541	24.5	249	26.9	272	26.3	1377	25.2%
Active	129	20.1	141	21.5	540	24.4	228	24.6	257	24.8	1295	23.7%
Deaths during follow-up	218	34.0	196	29.9	585	26.5	246	26.6	306	29.6	1551	28.4%
Lost weight in last 5 years (2HE)												
Diet	61	22.9	42	15.8	92	34.6	28	10.5	43	16.2	266	4.9%
Illness	28	21.7	7	5.4	51	39.5	17	13.2	26	20.2	129	2.4%
*WOMEN, N (row %)*	873 (12.7)		875 (12.7)		2596 (37.8)		1131 (16.5)		1393 (20.3)		6868	100.0%
WC at 1HE (cm)	87.2	11.4	82.6	10.5	80.0	9.5	79.1	9.7	79.6	9.2	81.0	10.2
WC at 2HE (cm)	78.8	10.7	78.9	10.5	80.1	9.6	82.8	9.7	88.0	10.0	81.9	10.5
WC change (cm)	−8.4	3.5	−3.7	0.8	0.1	1.4	3.7	0.7	8.4	3.3	0.8	5.6
Annual WC change (cm)	−2.4	1.2	−1.1	0.3	0.0	0.4	1.1	0.3	2.4	1.1	0.2	1.6
Age at 1HE (years)	58.7	9.0	58.5	9.2	58.0	8.9	57.4	8.6	57.5	8.5	58.0	8.8
Age at 2HE (years)	61.9	9.2	61.6	9.3	61.2	9.0	60.5	8.6	60.6	8.6	61.1	8.9
Age change (years)	3.1	0.8	3.2	0.8	3.1	0.8	3.1	0.8	3.2	0.8	3.2	0.8
Weight at 1HE (kg)	70.1	12.8	67.8	11.7	66.2	10.4	66.7	10.6	68.5	10.7	67.5	11.1
Weight at 2HE (kg)	67.7	12.1	67.3	11.7	67.3	10.8	69.2	11.2	73.0	12.0	68.9	11.6
Weight change (kg)	−2.4	4.8	−0.5	3.3	1.1	2.9	2.5	3.2	4.5	4.2	1.4	4.1
BMI at 1HE (kg/m^2^)	26.9	4.6	26.0	4.3	25.5	3.8	25.6	3.9	26.3	3.9	25.9	4.0
BMI at 2HE (kg/m^2^)	26.1	4.3	26.0	4.4	26.0	4.0	26.6	4.2	28.1	4.4	26.6	4.3
BMI change (kg/m^2^)	−0.8	1.8	−0.1	1.2	0.5	1.1	1.0	1.2	1.9	1.6	0.7	1.6
Education - 1HE												
None	291	33.3	321	36.7	983	37.9	402	35.5	528	37.9	2525	36.8%
O level and above	582	66.7	554	63.3	1613	62.1	729	64.5	865	62.1	4343	63.2%
Social class - 1HE												
Non-manual	537	61.5	558	63.8	1659	63.9	727	64.3	877	63.0	4358	63.4%
Manual	336	38.5	317	36.2	937	36.1	404	35.7	516	37.0	2510	36.6%
Smoking status - 1HE												
Current	94	10.8	72	8.2	224	8.6	100	8.8	128	9.2	618	9.0%
Former	290	33.2	287	32.8	794	30.6	329	29.1	469	33.7	2169	31.6%
Never	489	56.0	516	59.0	1578	60.8	702	62.1	796	57.1	4081	59.4%
Smoking status - 2HE												
Current	88	10.1	70	8.0	206	7.9	76	6.7	105	7.5	545	7.9%
Former	297	34.0	289	33.0	814	31.4	353	31.2	494	35.5	2247	32.7%
Never	488	55.9	516	59.0	1576	60.7	702	62.1	794	57.0	4076	59.4%
Physical activity - 1HE												
Inactive	234	26.8	236	27.0	622	24.0	245	21.7	369	26.5	1706	24.8%
Moderately inactive	282	32.3	275	31.4	874	33.7	390	34.5	457	32.8	2278	33.2%
Moderately active	220	25.2	206	23.5	631	24.3	291	25.7	328	23.6	1676	24.4%
Active	137	15.7	158	18.1	469	18.1	205	18.1	239	17.2	1208	17.6%
Deaths during follow-up	212	24.3	181	20.7	487	18.8	176	15.6	259	18.6	1315	19.2%
Menopausal status (2HE)												
Pre-menopausal	51	5.8	55	6.3	170	6.6	67	5.9	86	6.2	429	6.3%
Early peri-menopausal	26	3.0	34	3.9	80	3.1	63	5.6	48	3.5	251	3.7%
Late peri-menopausal (1–5 y)	149	17.1	168	19.2	504	19.4	213	18.8	288	20.7	1322	19.3%
Post-menopausal (> 5 y)	647	74.1	618	70.6	1842	71.0	788	69.7	971	69.7	4866	70.9%
Lost weight in last 5 years (2HE)												
Diet	179	23.6	108	14.3	233	30.8	94	12.4	143	18.9	757	11.0%
Illness	33	19.5	25	14.8	62	36.7	26	15.4	23	13.6	169	2.5%

Values are mean and SD or frequency and percentage

*1HE* 1st health examination, *2HE* 2nd health examination, *BMI* body mass index

### Main analyses: total and CVD mortality

At the end of a median (IQR) follow-up time of 16.4 (15.7, 17.2) years, 1551 deaths in men and 1315 deaths in women were recorded, 440 and 382 deaths from CVD, respectively. Total and CVD mortality HRs for men and women by WC change category are shown in Table 2. The addition of change in weight to the model strengthened the positive WCG-mortality associations (model 3), especially in men, where the findings for CVD mortality were stronger than for all-cause mortality, in those with a WCG > 5 cm (HR 1.84 and 1.51 respectively). Women with a WCG > 5 cm had a significantly higher hazard of all-cause mortality of 1.25 (CI 1.06–1.46) compared to WCM. No significant associations were found regarding changes in WC and CVD mortality in women. No significant associations were found for WCL and total or CVD mortality in the multi-variate adjusted models, in either men or women.

**Table 2:** Associations between total and CVD mortality in 5469 men and 6868 women and categories of change in waist circumference

	Categories of change in waist circumference (WC) (cm)				
	Loss > 5 cm	Loss > 2.5 and ≤ 5 cm	Loss or gain ≤2.5 cm	Gain > 2.5 and ≤ 5 cm	Gain > 5 cm
Men, N (%)	641 (11.7)	656 (12.0)	2211 (40.4)	926 (16.9)	1035 (18.9)
All-cause mortality					
Number of events	218 (34.0)	196 (29.9)	585 (26.5)	246 (26.6)	306 (29.5)
Model 1	** 1.28 (1.09–1.49)	1.07 (0.91–1.26)	Ref	1.04 (0.90–1.21)	* 1.19 (1.03–1.36)
Model 2	1.14 (0.97–1.34)	1.03 (0.87–1.21)	Ref	1.06 (0.91–1.23)	** 1.26 (1.10–1.46)
Model 3	0.93 (0.78–1.10)	0.95 (0.80–1.12)	Ref	1.15 (0.99–1.34)	*** 1.51 (1.29–1.75)
CVD mortality					
Number of events	64 (10.0)	62 (9.4)	153 (6.9)	66 (7.1)	95 (9.2)
Model 1	* 1.42 (1.06–1.90)	1.28 (0.95–1.72)	Ref	1.08 (0.81–1.44)	** 1.42 (1.10–1.84)
Model 2	1.22 (0.90–1.64)	1.21 (0.90–1.63)	Ref	1.09 (0.82–1.46)	** 1.53 (1.17–1.98)
Model 3	0.97 (0.70–1.34)	1.11 (0.82–1.49)	Ref	1.18 (0.88–1.58)	*** 1.84 (1.39–2.43)
Women, N (%)	873 (12.7)	875 (12.7)	2596 (37.8)	1131 (16.5)	1393 (20.3)
All-cause mortality					
Number of events	212 (24.3)	181 (20.7)	487 (18.8)	176 (15.6)	259 (18.6)
Model 1	** 1.26 (1.08–1.48)	1.03 (0.87–1.22)	Ref	0.86 (0.73–1.03)	1.09 (0.93–1.26)
Model 2	1.13 (0.95–1.33)	0.97 (0.82–1.16)	Ref	0.91 (0.76–1.08)	1.15 (0.98–1.34)
Model 3	1.03 (0.87–1.24)	0.93 (0.78–1.10)	Ref	0.95 (0.79–1.13)	** 1.25 (1.06–1.46)
CVD mortality					
Number of events	49 (5.6)	56 (6.4)	152 (5.8)	50 (4.4)	75 (5.4)
Model 1	0.91 (0.66–1.26)	0.97 (0.72–1.32)	Ref	0.81 (0.59–1.12)	1.04 (0.79–1.38)
Model 2	0.80 (0.57–1.11)	0.91 (0.67–1.24)	Ref	0.87 (0.63–1.20)	1.14 (0.86–1.52)
Model 3	0.77 (0.54–1.09)	0.89 (0.65–1.22)	Ref	0.87 (0.63–1.21)	1.15 (0.85–1.55)

***p < 0.001; **p < 0.01; *p < 0.05

Associations were assessed using Cox proportional hazards regression with a median follow-up from 2HE of 16 years. Results are hazard ratios and 95% confidence intervals, HR (95% CI)

Model 1: adjusted for age (continuous)

Model 2: Model 1 + further adjusted for BMI (continuous), waist circumference (continuous), smoking (categorical), physical activity (categorical), educational level (categorical) and social class (categorical), and menopausal status (categorical) in women

Model 3: Model 2 + further adjusted for weight change (continuous)

*2HE* 2nd health examination, *CVD* cardiovascular disease, *BMI* body mass index

### Subgroup analyses

HRs for all-cause mortality in men and women, adjusted as per model 3, grouped by confounder categories are shown in Tables 3 and 4 respectively. Participants with a WCG > 5 cm had a higher HR than those in the WCM category, irrespective of their WC categorisation at 1HE, although stronger associations were more evident in men. Men who had a WCG > 5 cm had significantly higher HRs, irrespective of whether they were < 65 years (HR 1.78 CI 1.40–2.26) or > 65 years (HR 1.38 (CI 1.13–1.68) of age at 1HE (Table 3) although a significant association was only found among women > 65 years (HR 1.35 (CI 1.10–1.67) (Table 4). Higher hazards were observed for participants with a WCG > 5 cm, irrespective of smoking status, with the exception of women who were former smokers, where a WCG between 2.5 and 5 cm had a significantly lower HR of 0.62 (CI 0.45–0.86) (Table 4). Irrespective of BMI, physical activity, educational level or social class categorisation at HE1, WCG > 5 cm was associated with a higher HR in both men and women (Tables 3 and 4 respectively). Almost 71% of women were classified as postmenopausal and a higher hazard was found in those with a WCG > 5 cm (Table 4). Even after excluding participants who died within 3 or 5 years of the 2HE, participants with a WCG > 5 cm had significantly higher HRs for all-cause mortality, than those in the WCM category.

**Table 3:** Cox multivariable-adjusted HRs after 16 years of follow-up for all-cause mortality in 5469 men. Results are given for stratified variables by WC change category

					Categories of change in waist circumference (WC) (cm)				
					loss > 5 cm	loss > 2.5 & ≤5 cm	loss or gain ≤2.5 cm	gain > 2.5 & ≤ 5 cm	gain > 5 cm
	N	%	Deaths	%	HR (95% CI)	HR (95% CI)		HR (95% CI)	HR (95% CI)
All	5469	100.0	1551	100.0	0.93 (0.78–1.10)	0.95 (0.80–1.12)	Ref	1.15 (0.99–1.34)	*** 1.51 (1.29–1.75)
By WC (1HE)									
< 94 cm	2515	46.0	604	38.9	0.93 (0.67–1.28)	1.20 (0.91–1.57)	Ref	1.04 (0.81–1.32)	* 1.28 (1.02–1.61)
≥ 94 & ≤ 102 cm	1854	33.9	520	33.5	0.93 (0.69–1.24)	0.79 (0.58–1.06)	Ref	* 1.34 (1.04–1.73)	*** 1.96 (1.50–2.55)
> 102 cm	1100	20.1	427	27.5	0.89 (0.66–1.20)	0.90 (0.67–1.21)	Ref	1.20 (0.88–1.65)	* 1.42 (1.02–1.99)
By age (1HE)									
< 65 y	3882	71.0	573	36.9	0.99 (0.73–1.34)	0.97 (0.73–1.29)	Ref	1.22 (0.95–1.57)	*** 1.78 (1.40–2.26)
≥ 65 y	1587	29.0	978	63.1	0.91 (0.74–1.13)	0.94 (0.77–1.15)	Ref	1.14 (0.94–1.38)	** 1.38 (1.13–1.68)
By smoking status (2HC)									
current	438	8.0	170	11.0	0.75 (0.45–1.23)	0.98 (0.60–1.58)	Ref	1.01 (0.61–1.68)	** 2.11 (1.32–3.36)
former	2970	54.3	983	63.4	0.91 (0.72–1.14)	0.89 (0.72–1.10)	Ref	1.15 (0.95–1.39)	*** 1.54 (1.28–1.87)
never	2061	37.7	398	25.7	1.17 (0.84–1.63)	1.08 (0.78–1.49)	Ref	1.26 (0.93–1.70)	1.22 (0.89–1.67)
By BMI (1HE)									
≥ 18.5 & < 25	1913	35.0	505	32.6	1.03 (0.75–1.41)	0.94 (0.70–1.26)	Ref	1.03 (0.79–1.34)	1.29 (0.99–1.69)
≥ 25 & < 30	2926	53.5	831	53.6	0.80 (0.63–1.02)	0.94 (0.75–1.17)	Ref	1.22 (0.99–1.50)	***1.69 (1.37–2.08)
≥ 30	630	11.5	215	13.9	1.26 (0.81–1.95)	0.89 (0.56–1.42)	Ref	1.38 (0.91–2.10)	1.34 (0.88–2.04)
By physical activity (1HC)									
inactive	1444	26.4	545	35.1	0.91 (0.68–1.21)	0.98 (0.74–1.30)	Ref	1.21 (0.93–1.57)	*** 1.72 (1.34–2.22)
mod inactive	1353	24.7	374	24.1	1.03 (0.72–1.49)	1.08 (0.79–1.47)	Ref	1.14 (0.82–1.58)	* 1.47 (1.07–2.03)
mod active	1377	25.2	336	21.7	0.86 (0.59–1.23	0.69 (0.47–1.01)	Ref	0.95 (0.69–1.30)	1.19 (0.86–1.65)
active	1295	23.7	296	19.1	0.88 (0.57–1.33)	1.01 (0.69–1.49)	Ref	1.22 (0.87–1.70)	* 1.50 (1.06–2.12)
By educational level (1HE)									
No qualifications	1444	26.4	537	34.6	0.90 (0.68–1.20)	1.03 (0.78–1.36)	Ref	0.90 (0.68–1.19)	*** 1.76 (1.36–2.28)
O level and above	4025	73.6	1014	65.4	0.96 (0.77–1.19)	0.90 (0.73–1.11)	Ref	** 1.29 (1.08–1.55)	** 1.38 (1.14–1.67)
By social class (1HE)									
Non-manual	3395	62.1	932	60.1	1.03 (0.82–1.29)	0.90 (0.73–1.11)	Ref	1.12 (0.92–1.37)	** 1.39 (1.14–1.69)
Manual	2074	37.9	619	39.9	0.81 (0.62–1.06)	1.04 (0.80–1.35)	Ref	1.17 (0.92–1.49)	*** 1.67 (1.31–2.13)
Excluding early deaths									
Excluding deaths < 3 y	5347	97.8	1429	92.1	0.90 (0.75–1.08)	0.94 (0.79–1.12)	Ref	1.16 (0.99–1.35)	*** 1.52 (1.29–1.78)
Excluding deaths < 5 y	5211	95.3	1293	83.4	0.94 (0.78–1.14)	0.94 (0.79–1.13)	Ref	1.13 (0.96–1.34)	*** 1.45 (1.23–1.72)

Adjusted for age, BMI, baseline WC, physical activity, smoking, educational level, social class and change in weight (except where the variable was used for stratification)

***p < 0.001; ** *p* < 0.01; * *p* < 0.05

**Table 4:** Cox multivariable-adjusted HRs after 16 years of follow-up for all-cause mortality in 6868 women. Results are given for stratified variables by WC change category

					Categories of change in waist circumference (WC) (cm)				
					loss > 5 cm	loss > 2.5 & ≤5 cm	loss or gain ≤2.5 cm	gain > 2.5 & ≤ 5 cm	gain > 5 cm
	N	%	Deaths	%	HR (95% CI)	HR (95% CI)		HR (95% CI)	HR (95% CI)
All	6868	100.0	1315	100.0	1.03 (0.87–1.24)	0.93 (0.78–1.10)	Ref	0.95 (0.79–1.13)	** 1.25 (1.06–1.46)
By WC (1HE)									
< 80 cm	3510	51.1	516	39.2	1.14 (0.82–1.57)	1.00 (0.75–1.33)	Ref	0.95 (0.73–1.23)	1.07 (0.83–1.38)
≥ 80 & ≤ 88 cm	1868	27.2	392	29.8	1.26 (0.92–1.73)	1.11 (0.81–1.51)	Ref	0.78 (0.54–1.11)	1.28 (0.96–1.71)
> 88 cm	1490	21.7	407	31.0	0.80 (0.60–1.07)	0.76 (0.55–1.03)	Ref	1.06 (0.76–1.47)	** 1.53 (1.11–2.11)
By age (1HE)									
< 65 y	5153	75.0	518	39.4	1.23 (0.92–1.64)	1.07 (0.80–1.43)	Ref	1.04 (0.80–1.36)	1.10 (0.85–1.43)
≥ 65 y	1715	25.0	797	60.6	0.92 (0.74–1.15)	0.83 (0.66–1.03)	Ref	0.89 (0.70–1.12)	** 1.35 (1.10–1.67)
By smoking status (2HC)									
current	545	7.9	127	9.7	1.30 (0.78–2.15)	0.81 (0.44–1.51)	Ref	1.26 (0.71–2.24)	1.15 (0.64–2.07)
former	2247	32.7	479	36.4	0.80 (0.60–1.08)	0.79 (0.60–1.06)	Ref	** 0.62 (0.45–0.86)	1.14 (0.88–1.48)
never	4076	59.3	709	53.9	1.15 (0.90–1.48)	1.05 (0.83–1.33)	Ref	1.13 (0.90–1.42)	* 1.32 (1.06–1.65)
By BMI (1HE)									
≥ 18.5 & < 25	3258	47.4	548	41.7	1.09 (0.82–1.43)	0.91 (0.69–1.19)	Ref	0.93 (0.71–1.20)	1.27 (0.98–1.64)
≥ 25 & < 30	2614	38.1	540	41.1	1.04 (0.79–1.38)	1.00 (0.76–1.30)	Ref	0.84 (0.62–1.13)	1.23 (0.96–1.58)
≥ 30	996	14.5	227	17.3	0.86 (0.57–1.32)	0.82 (0.52–1.29)	Ref	1.17 (0.78–1.75)	1.31 (0.88–1.96)
By physical activity (1HC)									
inactive	1706	24.8	501	38.1	1.14 (0.86–1.51)	0.97 (0.74–1.27)	Ref	0.97 (0.72–1.30)	1.06 (0.81–1.39)
mod inactive	2278	33.2	420	31.9	1.15 (0.83–1.58)	1.09 (0.79–1.50)	Ref	1.11 (0.82–1.50)	** 1.51 (1.14–2.00)
mod active	1676	24.4	247	18.8	0.67 (0.43–1.03)	0.74 (0.49–1.12)	Ref	0.70 (0.47–1.03)	1.14 (0.79–1.65)
active	1208	17.6	147	11.2	1.15 (0.70–1.92)	0.73 (0.42–1.27)	Ref	0.92 (0.54–1.54)	1.41 (0.86–2.32)
By educational level (1HE)									
No qualifications	2525	36.8	670	51.0	1.07 (0.84–1.38)	0.88 (0.69–1.12)	Ref	0.88 (0.68–1.12)	1.20 (0.95–1.50)
O level and above	4343	63.2	645	49.0	1.00 (0.78–1.29)	0.99 (0.77–1.27)	Ref	1.02 (0.80–1.31)	* 1.29 (1.03–1.63)
By social class (1HE)									
Non-manual	4358	63.5	848	64.5	1.00 (0.80–1.26)	0.99 (0.80–1.22)	Ref	0.80 (0.64–1.01)	** 1.30 (1.07–1.58)
Manual	2510	36.5	467	35.5	1.08 (0.81–1.44)	0.81 (0.59–1.10)	Ref	1.21 (0.92–1.59)	1.14 (0.85–1.51)
Menopausal status (2HC)									
Pre-menopausal	429	6.2	9	0.7	0.00 (0.00 -)	5.16 (0.75–35.62)	Ref	0.00 (0.00 -)	3.91 (0.61–24.96)
Early peri-menopausal (< 1 y)	251	3.7	10	0.8	0.00 (0.00 -)	0.38 (0.04–4.00)	Ref	0.52 (0.09–2.90)	0.77 (0.11–5.21)
Late peri-menopausal (1–5 y)	1322	19.2	68	5.2	* 2.39 (1.00–5.69)	1.79 (0.81–3.98)	Ref	** 2.84 (1.40–5.77)	1.51 (0.69–3.32)
Post-menopausal (> 5 y)	4866	70.9	1228	93.4	1.01 (0.84–1.21)	0.89 (0.74–1.06)	Ref	0.88 (0.74–1.06)	* 1.23 (1.04–1.45)
Excluding early deaths									
Excluding deaths < 3 y	6800	99.0	1247	94.8	1.02 (0.85–1.22)	0.94 (0.78–1.12)	Ref	0.96 (0.80–1.15)	** 1.23 (1.04–1.33)
Excluding deaths < 5 y	6713	97.7	1160	88.2	1.03 (0.86–1.2)	0.94 (0.78–1.13)	Ref	0.98 (0.82–1.18)	* 1.24 (1.04–1.48)

Adjusted for age, BMI, baseline WC, physical activity, smoking, educational level, social class, menopausal status and change in weight (except where the variable was used for stratification)

***p < 0.001; ** *p* < 0.01; * *p* < 0.05

As significant findings were evident for CVD mortality and men, but not women, in relation to changes in WC, we further explored these in subgroups, stratified by potential confounding characteristics, adjusted as per model 3 (Additional file 1: Table S2). Generally, a higher hazard was evident for all values of the confounding variables in men with a WCG; findings relating to WCL were inconsistent. In men who had a WCG > 5 cm, the strongest associations were found in those who were younger, current smokers, overweight, physically active, had no qualifications, had a manual job and a normal WC. Even after excluding participants who died within 3 or 5 years of the 2HE, men with a WCG had significantly higher HRs for CVD mortality, than those in the WCM category.

### Total and CVD mortality: WC changes in relation to weight changes

Weight change was strongly positively correlated with a change in WC in both men (r = 0.60) and women (*r* = 0.57) (*P* < 0.001). We also explored the association of category changes in WC and total mortality in combination with category changes in weight; the interaction between WC change categories and weight change categories was not found to be significant (*p* = 0.29 for men and 0.82 for women). No significant interaction was found between WC change categories and weight change categories with regard to CVD mortality (*p* = 0.71 for men and 0.52 for women).

**Fig 2:**
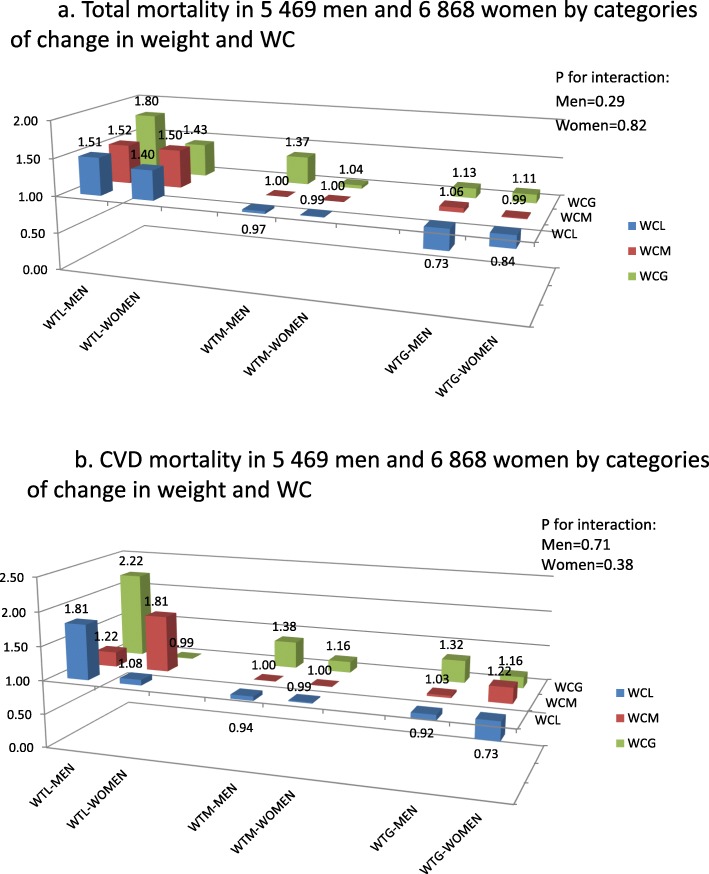
a Total mortality in 5 469 men and 6 868 women by categories of change in weight and WC, b. CVD mortality in 5 469 men and 6 868 women by categories of change in weight and WC

Figure 2a illustrates the associations between category changes in WC and weight and total mortality, adjusted for age, BMI, baseline WC, physical activity, smoking, educational level, social class and change in weight, and menopausal status in women. Similar trends in both men and women were generally evident. In men, significantly higher HRs were observed for all WTL categories, irrespective of whether men were categorised as WCL, WCM or WCG, as well as for WCG and WTM (HR 1.37 (CI 1.17–1.61)), compared to those in the WTM and WCM category. In women, significantly higher HRs were observed for total mortality for 2 categories – WTL and WCL (HR 1.40 (CI 1.14–1.70)) and WTL and WCM (HR 1.50 (CI 1.16–1.95)), compared to the reference category of WTM and WCM.

Figure 2b illustrates the similar associations with CVD mortality, adjusted for age, BMI, baseline WC, physical activity, smoking, educational level, social class and change in weight, and menopausal status in women. In men, significantly higher HRs were found in the WTL and WCL (HR 1.81 (CI 1.32–2.49)), WTL and WCG (HR 2.22 (CI 1.03–4.82)), and WTM and WCG (HR 1.38 (CI 1.02–1.88)) categories, compared to the reference category of WTM and WCM. A significant association for CVD mortality in women was only found in the WTL and WCM category (HR 1.81 (CI 1.15–2.85)). In all analyses, the category with the lowest HR was WTG and WCL.

## Discussion

### Summary of main findings

In this study of 12,337 middle-aged and elderly men and women, a WCG of more than 5 cm over approximately 4 years was associated with higher total mortality in both men and women and higher CVD mortality in men over the next 16 years of follow-up. Particularly among men, the associations were also evident after stratification for age, smoking, BMI, physical activity, educational level, social class and after the exclusion of deaths within the first 5 years of follow-up. Associations for WCL and mortality were inconsistent.

### Strengths and limitations

The major strengths of this study include its large population of free-living, middle-aged and elderly men and women, its prospective design, and the long follow-up time. Additionally, WC, weight and height were objectively measured, not self-reported, and information on a large number of factors associated with WC and weight was available. To minimize the possibility of reverse causality, that is, participants who were sub-clinically ill were more likely to lose weight and WC which would have resulted in a higher mortality risk; participants were excluded with self-reported cancer or CVD at both time-points, as were those who had a BMI < 18.5 kg/m^2^. Those who died within the first 3 and 5 years were also excluded in our subgroup analyses. Nevertheless, we cannot completely rule out the possibility of some effect of reverse causality and/or residual confounding.

The main limitations of our study include self-reported disease history, healthy volunteer bias and attrition. The prevalence of self-reported CVD was lower in those who also attended the 2HE, as was the percentage of current smokers and physically inactive participants, which may be indicative of healthier individuals being more likely to return for a follow up assessment (Additional file 1: Table 1). Additionally, the percentage of deaths that occurred was lower in both men and women, after exclusion criteria were applied; the percentage of deaths was highest in those who only attended the 1HE. It is possible that some individuals in our study had undiagnosed/sub-clinical conditions such as digestive disorders, or various psychosocial diseases, such as depression, that they did not report, resulting in weight and potentially WC loss and subsequent death, and possibly an overestimation of the association [39]. The more frail participants may have been excluded and/or have not returned for the 2HC and therefore be under-represented in our analyses. However, it is also conceivable that some of the participants in the weight and WC loss categories were pre-frail at 2HE. A further limitation is the inability to account for all behaviour changes during follow-up, including physical activity, which may have had effects on the observed associations.

### Comparison of total and CVD mortality with other studies

The highest HRs for both total and CVD mortality in men, which were statistically significant, were observed in the WTL and WCG category, although this was a small category, consisting of only 35 participants and 19 events. In women, the highest HR was found in the WTL and WCM category, for both total and CVD mortality. The category with the lowest HR in both men and women, for both total and CVD mortality was the weight gain and WC loss category, although this was not statistically significant. The findings that WTL, in combination with changes in WC, was associated with the highest HRs, agrees with our previous study, which found that weight loss of more than 2.5 kg over approximately 4 years is associated with a higher mortality over 15 years of follow-up [31] in this middle-aged and elderly population.

Our findings that WCG is associated with higher all-cause mortality are in agreement with a number of previous publications. A recent pooled sample of 2492 Danish and Swedish women found that WCG over 6 years was associated with increased total and CVD mortality [23]. We did not find significant associations with WCG and CVD mortality in women; statistical power was more limited due to the lower number of CVD deaths in women in our study. In the study of Danish and Swedish women, associations were particularly strong for women with normal weight and for ever-smokers. In our analyses, we found that WCG > 5 cm had a similar HR for all BMI categories and only never smokers had a statistically higher HR. The differences in findings may be due to the characteristics of the 2 cohorts; our cohort of women was older, had a higher mean BMI and a lower percentage of ever-smokers. A prospective study of 26,625 healthy middle-aged men and women has also shown that increases in WC over a 5.3 year period were positively associated with mortality in men and women over 6.7 years of follow-up; the HR was 1.09 (1.02, 1,16) per 5 cm for both men and women combined, after adjusting for baseline BMI, WC and change in BMI [24].

However, results from the Melbourne Collaborative Cohort Study of 21,298 men and women aged 40–69 years at baseline found that WCL over approximately 12 years was associated with an increased risk of all-cause mortality (HR: 1.26; 95% CI: 1.09–1.47) over 7.7 years of follow-up, especially in older adults, compared to those who had minimal changes, but did not find an association with WCG [25]. It is possible that the shorter time period of less than 4 years between our WC measurements may partially explain our lack of associations with WCL and mortality. However, in a study of older adults aged 70–77 years, WCL over a 3 year period (≥3.1 cm) was significantly associated with an all-cause mortality risk over 20 years of follow-up of 1.52 (95% CI: 1.01–2.31), which was comparable to their weight loss finding, in agreement with our previous results [31]; no significant associations were found for WCG and all-cause mortality, or changes in WC and CVD mortality [40]. No significant associations were found with self-reported changes in WC over a 12 years and concurrent mortality, after 9 years of follow-up, in a large, prospective study among 23,254 Swedish women [26], although the cohort was relatively young (median age of 52 years at follow-up), with a relatively low number of deaths (*N* = 570) during follow-up, of which 79 were attributable to CVD. A smaller study of 1138 older adults, predominantly female, from the Bambuí (Brazil) Cohort Study of Aging [27] did not find any associations with WC change and mortality; the relatively short time interval of 3 years between measurements, in addition to the follow-up period of 8 years may have contributed to the lack of findings. No associations were found for WC change over 3.6 years and mortality in a study of 1805 Iranian men, aged ⩾30 years [28]; the limited number of deaths (*N* = 88) during the relatively short-term follow-up of 6.6 years resulted in limited power to assess the effect of changes in WC for mortality events.

### Changes in WC and weight and plausible mechanisms

Weight, particularly in older populations, is an indicator encompassing not just fat but also bone and muscle mass and these different components may relate to health in different directions; thus, weight loss in later life may be an indicator of increasing frailty. Body fat distribution changes with age, with a reduction in subcutaneous fat and an increase in central adiposity [41]. The accumulation of body fat in the abdominal region has been shown to be associated with a number of adverse health outcomes such as diabetes, metabolic syndrome, CVD and all-cause mortality, independently of BMI [12, 42–44]. Adipose tissue has an important role to play in numerous metabolic and endocrine functions, including the expression and secretion of inflammatory cytokines, such as leptin, adiponectin, and interleukin 6 (IL-6), which are important regulatory factors of energy intake and inflammatory responses [45]. In a study of 20 abdominally obese, older women, hyperinsulinemia was positively associated with adipose IL-6 gene expression, but negatively associated with adipose adiponectin expression [46]. A 20-week weight loss program in obese, older women decreased leptin production in both gluteal and abdominal adipose tissue, but only increased adiponectin production from abdominal adipose tissue [47]. These results highlight the importance that regional adipose tissue hormone/cytokine production may play in mechanisms of metabolic complications associated with abdominal obesity and the importance of improving body fat distribution, through appropriate exercise and diet programs [48].

### Public health considerations

Obesity is a complex condition, for which there are numerous risk factors, many of which are modifiable [49, 50]. Attention needs to be focussed on various levels of policy strategies for its prevention [51, 52]. Aa a number of studies have shown that WC is increasing at a faster rate than BMI or body weight [53–55], current recommendations, based on observational cohort studies, including findings from this publication, should advocate prevention of WC gain into adulthood, and weight and WC stability from midlife onwards.

## Conclusion

In summary, an increase in WC of more than 5 cm over approximately 4 years, with little weight gain, is significantly associated with higher total mortality in both men and women and higher CVD mortality in men over the next 16 years of follow-up in this population-based cohort study of 12,337 middle-aged and elderly men and women. These findings are in marked contrast to our earlier observation that weight loss is associated with increased mortality risk [31]. The apparently paradoxical observations suggest that WC may be a better indicator of the adverse health consequence of obesity in later life than weight. Interventions targeted at fat distribution and focusing on preventing increase in central adiposity rather than lowering weight per se in later life may be more likely to have health benefits.

## Supplementary information

**Additional file 1.** Supplementary information. Baseline characteristics of EPIC-Norfolk men and women who attended 1HE, and those who attended both 1HE and 2HE, before and after exclusion criteria were applied. Cox multivariable-adjusted HRs after 16 years of follow-up for CVD mortality in 5469 men.

## Data Availability

The datasets analysed during the current study are available from the corresponding author on reasonable request.

## Abbreviations

1HE: Baseline health examination
2HE: Second health examination
BMI: Body mass index
CVD: Cardiovascular disease
EPIC: European prospective investigation into Cancer and nutrition
WC: Waist circumference
WCG: Waist circumference gain
WCL: Waist circumference loss
WCM: Waist circumference maintenance
WTG: Weight gain
WTL: Weight loss
WTM: Weight maintenance
