# Supplementary material for: Changes in waist circumference and risk of all-cause and CVD mortality: results from the European Prospective Investigation into Cancer in Norfolk (EPIC-Norfolk) cohort study
Source: BMC Cardiovasc Disord. 2019 Oct 28;19:238. doi: 10.1186/s12872-019-1223-z (PMC6819575; doi:10.1186/s12872-019-1223-z)
Supplement: Supplementary file 1 — Additional file 1. Supplementary information. Baseline characteristics of EPIC-Norfolk men and women who attended 1HE, and those who attended both 1HE and 2HE, before and after exclusion criteria were applied. Cox multivariable-adjusted HRs after 16 years of follow-up for CVD mortality in 5469 men. [file 12872_2019_1223_MOESM1_ESM.docx]

**Table S1** Baseline characteristics of EPIC-Norfolk men and women who attended 1HE, and those who attended both 1HE and 2HE, before and after exclusion criteria were applied. Continuous variables are Mean (SD) and categorical variables are N (%).

|  |  | **Men** |  |  |  | **Women** |  |
| --- | --- | --- | --- | --- | --- | --- | --- |
|  | 1HE  N=11 607 | 1HE and 2HE  N=6 582 | 1HE and 2HE with exclusions^a^  N=5 469 |  | 1HE  N=14 032 | 1HE and 2HE  N=8 446 | 1HE and 2HE with exclusions^a^  N=6 868 |
| Waist, cm | 95.8 (9.8) | 95.3 (9.4) | 95.1 (9.3) |  | 82.2 (10.9) | 81.2 (10.4) | 81.0 (10.2) |
| Weight, kg | 80.4 (11.5) | 80.1 (11.0) | 80.2 (10.9) |  | 67.9 (11.8) | 67.4 (11.3) | 67.5 (11.1) |
| Age, years | 59.6 (9.3) | 59.8 (8.9) | 59.0 (8.9) |  | 58.9 (9.3) | 58.4 (8.9) | 57.9 (8.8) |
| BMI, kg/m2 | 26.5 (3.3) | 26.4 (3.2) | 26.3 (3.1) |  | 26.2 (4.4) | 25.9 (4.1) | 25.9 (4.0) |
| Smoking status |  |  |  |  |  |  |  |
| Current | 1 405 (12.2) | 616 (9.4) | 507 (9.3) |  | 1 579 (11.4) | 764 (9.1) | 618 (9.0) |
| Former | 6 284 (54.5) | 3 563 (54.5) | 2 898 (53.0) |  | 4 477 (32.2) | 2 632 (31.4) | 2 169 (31.6) |
| Never | 3 837 (33.3) | 2 355 (36.0) | 2 064 (37.7) |  | 7 837 (56.4) | 4 977 (59.4) | 4 081 (59.4) |
| Physical activity |  |  |  |  |  |  |  |
| Inactive | 3 586 (30.9) | 1 826 (27.7) | 1 444 (26.4) |  | 4 277 (30.5) | 2 226 (26.4) | 1 706 (24.8) |
| Moderately inactive | 2 858 (24.6) | 1 649 (25.1) | 1 353 (24.7) |  | 4 493 (32.0) | 2 760 (32.7) | 2 278 (33.2) |
| Moderately active | 2 660 (22.9) | 1 632 (24.8) | 1 377 (25.2) |  | 3 116 (22.2) | 2 018 (23.9) | 1 676 (24.4) |
| active | 2 502 (21.6) | 1 475 (22.4) | 1 295 (23.7) |  | 2 146 (15.3) | 1 442 (17.1) | 1 208 (17.6) |
| Social class |  |  |  |  |  |  |  |
| Non-manual | 6 657 (58.4) | 4 034 (62.2) | 3 395 (62.1) |  | 8 394 (61.4) | 5 304 (64.0) | 4 358 (63.4) |
| Manual | 4 744 (41.6) | 2 451 (37.8) | 2 074 (37.9) |  | 5 274 (38.6) | 2 978 (36.0) | 2 510 (36.6) |
| Educational level |  |  |  |  |  |  |  |
| No qualifications | 3 534 (30.5) | 1 801 (27.4) | 1 444 (26.4) |  | 5 920 (42.2) | 3 169 (37.5) | 2 525 (36.8) |
| O level and above | 8 064 (69.5) | 4 779 (72.6) | 4 025 (73.6) |  | 8 103 (57.8) | 5 272 (62.5) | 4 343 (63.2) |
| Self-reported diseases |  |  |  |  |  |  |  |
| CVD (yes) | 798 (6.9) | 394 (6.0) |  |  | 324 (2.3) | 160 (1.9) |  |
| Cancer (yes) | 450 (3.9) | 233 (3.5) |  |  | 960 (6.8) | 568 (6.7) |  |
| Deaths | 4 285 (36.9) | 2 196 (33.4) | 1 551 (28.4) |  | 3 754 (26.8) | 1 820 (21.6) | 1 315 (19.2) |
| WC change categories |  |  |  |  |  |  |  |
| loss >5 cm |  | 774 (11.8) | 641 (11.7) |  |  | 1 116 (13.2) | 873 (12.7) |
| loss >2.5 & ≤5 cm |  | 788 (12.0) | 656 (12.0) |  |  | 1 085 (12.8) | 875 (12.7) |
| Reference |  | 2 662 (40.4) | 2 211 (40.4) |  |  | 3 152 (37.3) | 2 596 (37.8) |
| gain >2.5 & ≤5 cm |  | 1 089 (16.6) | 926 (16.9) |  |  | 1 383 (16.4) | 1 131 (16.5) |
| gain >5 cm |  | 1 269 (19.3) | 1 035 (18.9) |  |  | 1 710 (20.2) | 1 393 (20.3) |
|  |  |  |  |  |  |  |  |

^a^ self-reported cancer and CVD, missing data on weight, height, waist, smoking status, social class, educational level, BMI < 18.5 kg/m^2^ and menopausal status in women

*BMI* body mass index, *1HE* 1st health examination, *2HE* 2nd health examination

**Table S2**. Cox multivariable-adjusted HRs after 16 years of follow-up for CVD mortality in 5 469 men. Results are given for stratified variables by WC change category.

|  |  |  |  |  |  | **Categories of change in waist circumference (WC) (cm)** | | | | |
| --- | --- | --- | --- | --- | --- | --- | --- | --- | --- | --- |
|  |  |  |  |  |  | loss > 5 cm | loss > 2.5 & ≤5 cm | loss or gain ≤ 2.5 cm | gain > 2.5 & ≤ 5 cm | gain > 5 cm |
|  |  | N | % | Deaths | % | HR (95% CI) | HR (95% CI) |  | HR (95% CI) | HR (95% CI) |
|  |  |  |  |  |  |  |  |  |  |  |
|  | All | 5 469 | 100.0 | 440 | 100.0 | 0.97 (0.70 - 1.34) | 1.11 (0.82 - 1.49) | Ref | 1.18 (0.88 - 1.58) | *** 1.84 (1.39 - 2.43) |
|  |  |  |  |  |  |  |  |  |  |  |
| By WC (1HE) | |  |  |  |  |  |  |  |  |  |
|  | < 94 cm | 2515 | 46.0 | 158 | 35.9 | 0.80 (0.41 - 1.56) | 1.48 (0.89 - 2.46) | Ref | 1.28 (0.80 - 2.05) | * 1.57 (1.00 - 2.46) |
|  | ≥ 94 & ≤ 102 cm | 1854 | 33.9 | 138 | 31.4 | 0.94 (0.53 - 1.68) | 0.94 (0.54 - 1.64) | Ref | 1.31 (0.77 - 2.22) | *** 2.66 (1.62 – 4.35) |
|  | > 102 cm | 1100 | 20.1 | 144 | 32.7 | 1.07 (0.64 - 1.78) | 1.03 (0.62 - 1.72) | Ref | 1.09 (0.62 - 1.91) | 1.56 (0.90 – 2.70) |
|  |  |  |  |  |  |  |  |  |  |  |
| By age (1HE) | |  |  |  |  |  |  |  |  |  |
|  | <65 y | 3882 | 71.0 | 134 | 30.5 | 1.39 (0.74 - 2.60) | * 1.80 (1.03 - 3.14) | Ref | * 1.89 (1.09 - 3.26) | *** 3.38 (2.03 - 5.64) |
|  | ≥65 y | 1587 | 29.0 | 306 | 69.5 | 0.89 (0.60 - 1.30) | 0.93 (0.64 - 1.34) | Ref | 1.02 (0.71 - 1.44) | * 1.46 (1.04 -2.05) |
|  |  |  |  |  |  |  |  |  |  |  |
| By smoking status (2HC) | |  |  |  |  |  |  |  |  |  |
|  | current | 438 | 8.0 | 55 | 12.5 | 1.33 (0.56 - 3.18) | 1.06 (0.42 - 2.67) | Ref | 1.23 (0.49 - 3.09) | ** 3.10 (1.39 - 6.94) |
|  | former | 2970 | 54.3 | 273 | 62.0 | 0.82 (0.53 - 1.27) | 1.03 (0.70 - 1.51) | Ref | 1.28 (0.89 - 1.82) | *** 1.69 (1.18 - 2.43) |
|  | never | 2061 | 37.7 | 112 | 25.5 | 1.32 (0.72 - 2.45) | 1.36 (0.75- 2.47) | Ref | 0.97 (0.51 - 1.84) | 1.70 (0.98 - 2.94) |
|  |  |  |  |  |  |  |  |  |  |  |
| By BMI (1HE) | |  |  |  |  |  |  |  |  |  |
|  | ≥ 18.5 & < 25 | 1913 | 35.0 | 137 | 31.1 | 1.09 (0.60 - 1.99) | 1.15 (0.67 - 1.99) | Ref | 1.19 (0.71 - 2.01) | 1.60 (0.96 - 2.66) |
|  | ≥ 25 & < 30 | 2926 | 53.5 | 220 | 50.0 | 0.80 (0.50 - 1.30) | 1.09 (0.71 - 1.67) | Ref | 1.24 (0.81 - 1.90) | ***2.37 (1.60 - 3.51) |
|  | ≥ 30 | 630 | 11.5 | 83 | 18.9 | 1.20 (0.59 - 2.44) | 1.17 (0.58 - 2.36) | Ref | 1.22 (0.62 - 2.41) | 1.28 (0.63 - 2.59) |
|  |  |  |  |  |  |  |  |  |  |  |
| By physical activity (1HC) | |  |  |  |  |  |  |  |  |  |
|  | inactive | 1444 | 26.4 | 160 | 36.4 | 0.65 (0.38 - 1.14) | 0.97 (0.59 - 1.60) | Ref | 0.87 (0.53 - 1.45) | 1.56 (0.98 - 2.47) |
|  | mod inactive | 1353 | 24.7 | 101 | 23.0 | 1.83 (0.97 - 3.44) | 1.28 (0.71 - 2.31) | Ref | 1.08 (0.56 - 2.11) | 1.48 (0.78 - 2.82) |
|  | mod active | 1377 | 25.2 | 93 | 21.1 | 0.84 (0.39 - 1.81 | 1.12 (0.56 - 2.22) | Ref | 1.80 (0.98 - 3.28) | * 2.16 (1.16 - 4.00) |
|  | active | 1295 | 23.7 | 86 | 19.5 | 1.08 (0.51 - 2.30) | 1.07 (0.51 - 2.26) | Ref | 1.35 (0.70 - 2.63) | ** 2.86 (1.57 - 5.23) |
|  |  |  |  |  |  |  |  |  |  |  |
| By educational level (1HE) | |  |  |  |  |  |  |  |  |  |
|  | No qualifications | 1444 | 26.4 | 150 | 34.1 | 0.74 (0.41 - 1.32) | 1.21 (0.73 - 2.01) | Ref | 1.09 (0.65 - 1.83) | ** 2.01 (1.25 - 3.25) |
|  | O level and above | 4025 | 73.6 | 290 | 65.9 | 1.13 (0.77 - 1.68) | 1.06 (0.72 - 1.54) | Ref | 1.23 (0.86 - 1.77) | ** 1.74 (1.23 - 2.46) |
|  |  |  |  |  |  |  |  |  |  |  |
| By social class (1HE) | |  |  |  |  |  |  |  |  |  |
|  | Non-manual | 3395 | 62.1 | 270 | 61.4 | 1.12 (0.75 - 1.69) | 0.95 (0.64 - 1.40) | Ref | 0.92 (0.62 - 1.36) | * 1.47 (1.03 - 2.09) |
|  | Manual | 2074 | 37.9 | 170 | 38.6 | 0.78 (0.45 - 1.35) | 1.44 (0.88 - 2.33) | Ref | * 1.70 (1.08 - 2.69) | *** 2.56 (1.62 - 4.06) |
|  |  |  |  |  |  |  |  |  |  |  |
| Excluding early deaths | |  |  |  |  |  |  |  |  |  |
|  | Excluding deaths < 3y | 5347 | 97.8 | 405 | 92.0 | 0.98 (0.69 - 1.37) | 1.14 (0.83 - 1.55) | Ref | 1.20 (0.88 - 1.62) | *** 1.80 (1.34 - 2.41) |
|  | Excluding deaths < 5y | 5211 | 95.3 | 357 | 81.1 | 1.05 (0.73 - 1.50) | 1.08 (0.78 - 1.52) | Ref | 1.08 (0.78 - 1.50) | ** 1.64 (1.20 - 2.25) |

Adjusted for age, BMI, baseline WC, physical activity, smoking, educational level, social class and change in weight (except where the variable was used for stratification)

***p<0.001; ** p<0.01; * p<0.05
